# Higher nodal yield with robot-assisted pelvic lymph node dissection for bladder cancer compared to laparoscopic dissection: implications for more accurate staging

**DOI:** 10.1080/2090598X.2020.1824570

**Published:** 2020-10-01

**Authors:** Amandeep Arora, Felipe Pugliesi, Ahmed S. Zugail, Marco Moschini, Cristiano Pazeto, Petr Macek, Armando Stabile, Camille Lanz, Nathalie Cathala, Mostefa Bennamoun, Rafael Sanchez-Salas, Xavier Cathelineau

**Affiliations:** a Department of Urology, Institut Mutualiste Montsouris, Paris, France; b Department of Urology, Lokmanya Tilak Municipal Medical College and General Hospital, Mumbai, India; c Division of Urology, Men’s Health Centre, Hospital Brigadeiro, Sao Paulo, Brazil; d Department of Urology, Faculty of Medicine in Rabigh, King Abdulaziz University, Jeddah, Saudi Arabia; e Department of Urology, Luzerner Kantonsspital, Luzern, Switzerland; f Department of Urology, Urological Research Institute, Vita-Salute University, San Raffaele Scientific Institute, Milan, Italy; g Department of Medical Oncology, Institut Mutualiste Montsouris, Paris, France

**Keywords:** Bladder cancer, pelvic lymph node dissection, robotic, laparoscopic, radical cystectomy

## Abstract

**Objectives:**

To compare the lymph node (LN) yield and adequacy of laparoscopic pelvic lymph node dissection (L-PLND) and robot-assisted PLND (R-PLND), as PLND is a fundamental component of radical cystectomy (RC) for bladder cancer (BCa), where a positive status is the most powerful predictor of disease recurrence and survival.

**Patents and methods:**

We retrospectively reviewed patients undergoing RC with PLND for BCa from January 2007 to July 2019 and grouped them in to L- and R-PLND. Until 2011, patients underwent a standard PLND (S-PLND) with the cranial limit as bifurcation of common iliac artery. Since 2012, an extended PLND (E-PLND) up to aortic bifurcation has been performed. An adequate S- and E-PLND were defined as those that yielded at least 10 and 16 LNs, respectively. The groups were compared for LN yield and adequacy of PLND.

**Results:**

During the study period, 305 patients underwent minimally invasive RC in our centre, of which 274 (89.8%) underwent a concomitant PLND (98 L-PLND, 176 R-PLND). R-PLND resulted in a significantly greater median LN yield compared to L-PLND, both in the S-PLND (16 vs 11, *P* < 0.001) and the E-PLND (19 vs 14, *P* < 0.001) eras. Also, a significantly higher proportion of patients in the R-PLND group had an adequate PLND compared to the L-PLND group. Surgical approach to PLND (R- vs L-PLND) was the only variable that was significantly associated with an adequate PLND on both univariable (odds ratio [OR] 1.860, 95% confidence interval [CI] 1.114–3.105; *P* = 0.01) and multivariable (OR 2.109, 95% CI 1.222–3.641; *P* = 0.007) analyses.

**Conclusion:**

R-PLND leads to a higher LN yield and a greater probability of an adequate PLND compared to L-PLND for both standard and extended templates. Therefore, the robot-assisted approach would lead to more accurate staging following RC with PLND.

## Introduction

About 25–30% of patients with bladder cancer (BCa) present with muscle-invasive disease. Radical cystectomy (RC), following neoadjuvant chemotherapy (NAC), is the standard of care for non-metastatic muscle-invasive bladder cancer (MIBC) and also for high-risk recurrent non-muscle-invasive disease [1]. Incidence of pelvic lymph node (LN) metastasis correlates with the T stage of the disease and ranges from 25% in T2 to 40–45% in T3/4 disease [2]. This makes pelvic LN dissection (PLND) an essential component of RC. A PLND is the most accurate method for LN staging of the disease [3,4]. This staging information helps to prognosticate patients for their recurrence-free rates and guides further decision-making regarding use of adjuvant chemotherapy/immunotherapy [5]. Also, there is increasing evidence for the therapeutic benefit of an extended PLND (E-PLND) in LN-negative as well as a subset of LN-positive patients (those with ≥pT3 disease) [6].

Although the role of PLND is well established, there seems to be no consensus as to what constitutes an ‘adequate PLND’. Parameters such as the number of dissected LNs and number of positive LNs (tumour burden) are used as surrogate markers of adequacy. Results from the Surveillance, Epidemiology and End Results (SEER) database showed that the dissection of at least 10–14 LNs during RC was an important prognostic factor [7]. Also, reports indicate that removal of a higher number of LNs is associated with improved survival following RC [8–10].

A RC with PLND is being increasingly performed via a minimally invasive approach. Despite reports of technical feasibility and similar complication rates compared to open surgery, there has been some scepticism about the adequacy of minimally invasive PLND. While most studies on laparoscopic PLND (L-PLND) report a lesser LN yield compared to open surgery, some have shown a comparable yield [11–13]. The initial reports with robot-assisted PLND (R-PLND) reported a lower yield; but with increasing experience and better ergonomics of the robotic systems, recent series report LN yields that are comparable to that in open RC [14–16].

Although laparoscopic RC with L-PLND has not been widely adopted, it is still a viable alternative to robot-assisted RC when availability of the robotic platform and cost constraints are an issue. However, data comparing L- and R-PLND are sparse [17–19]. At our institute we have been performing both laparoscopic and robot-assisted RC with PLND for our patients with BCa. In the present study, we aimed to compare L- and R-PLND with regards to LN yield and adequacy.

## Patients and methods

### Population

Our Institutional Review Board approved the study. A prospectively maintained institutional RC registry was searched for clinically non-metastatic BCa. From January 2007 to July 2019, 305 patients underwent RC for non-metastatic BCa at our institute, of which 274 patients (89.8%) underwent a bilateral PLND. Of these, 98 (35.7%) patients underwent L-PLND (L-PLND group), while the remaining 176 (64.3%) underwent R-PLND (R-PLND group). Patients with urothelial cancers, as well as those with histological variants were included.

Data were collected for age, gender, height, weight, body mass index (BMI), American Society of Anesthesiologists (ASA) score, age-adjusted Charlson Comorbidity Index (ACCI), whether NAC was received (for patients undergoing RC for urothelial MIBC), histological type of malignancy, pathological T and N stages, number of LNs resected (LN yield), number of positive LNs, carcinoma *in situ* (CIS), and margin positivity. Male patients underwent either a radical cystoprostatectomy or a prostate-sparing cystectomy, while females underwent an anterior pelvic exenteration. Up to 2011, patients underwent a ‘standard’ PLND (S-PLND) limited by the common iliac bifurcation superiorly, Cooper’s ligament inferiorly, genitofemoral nerve laterally and obturator nerve medially. From 2012 onwards, an E-PLND was performed with its cranial limit being the aortic bifurcation. LN packets were placed in a common bag. Either the right or left packet was clip identified for pathological information. The right-side packet included the presacral dissection if this was performed. An adequate S- and E-PLND were defined as those which yielded at least 10 and 16 LNs, respectively. These numbers were chosen based on previous studies that looked at the minimum number of LNs to be removed for an optimal dissection [7,20–22].

The L- and R-PLND groups were compared for LN yield, number of positive LNs and adequacy of PLND. This comparison was done for two separate time periods: the S-PLND period from 2007 to 2011 and the E-PLND period from 2012 to 2019. Also, to identify predictors of LN yield, a multivariable logistic regression model was developed with predictive variables including age, BMI, use of NAC, surgical approach (R- vs L-PLND) and pathological T-stage.

Our techniques of laparoscopic RC (LRC) and robot-assisted RC (RRC) with PLND have been previously described [23,24]. The procedures were performed by one of seven surgeons. Every surgeon was fellowship trained with sound experience in minimally invasive uro-oncological procedures, and performed both LRCs and RRCs. All pathological specimens were analysed by a team of dedicated uro-pathologists.

### Statistical analysis

The unpaired Student’s *t*-test, Mann–Whitney *U*-test and chi-square test were used to compare the statistical significance of differences in means, medians and proportions, respectively. Univariable and multivariable logistic regression analyses tested whether the surgical approach to PLND was an independent predictor of LN adequacy. Statistical significance was considered at *P* < 0.05. Statistical analyses were performed using the Statistical Package for the Social Sciences (SPSS®), version 20.0 (IBM Corp., Armonk, NY, USA).

## Results

### Preoperative and histopathology

Table 1 summarises the preoperative characteristics and histopathological features of the L- and R-PLND groups. The two cohorts were comparable for age, gender, BMI and the proportion of patients undergoing RC for very-high-risk non-MIBC (NMIBC) and MIBC. Patients in the R-PLND group had a higher ASA score (*P* < 0.001), higher ACCI (*P* = 0.004) and a higher proportion of use of NAC (83.5% vs 52%, *P* < 0.001) than those undergoing LRC. Pathological features including the T-stage, N-stage, CIS and proportion of patients with urothelial histology were similar across the two groups.

**Table 1.** Preoperative and histopathological characteristics of 274 patients who underwent minimally invasive RC with PLND from January 2007 to July 2019

Variable	Overall population (*N* = 274; 100%)	L-PLND (*n* = 98; 36%)	R-PLND (*n* = 176; 64%)	*P*
Age, years, median (IQR)	66 (61–73)	65 (60–72)	67 (62–73)	0.08
Gender, *n* (%) Male Female	239 (87) 35 (13)	82 (83) 16 (17)	157 (89) 19 (11)	0.2
BMI, kg/m^2^, mean (SD)	26.05 (4.33)	26.28 (4.79)	25.93 (4.07)	0.5
History of smoking, *n* (%) Non-smoker Current Past	48 (17.5) 108 (39.4) 88 (32)	14 (14.2) 34 (34.6) 29 (29.5)	34 (19.3) 74 (42) 59 (33.5)	0.9
ASA Score, *n* (%) I II III	47 (17) 170 (62) 39 (14)	29 (29.5) 52 (53) 4 (4)	18 (10.2) 118 (67) 35 (19.8)	<0.001
ACCI, *n* (%) 2–4 5–6 >6	119 (43.4) 96 (35) 46 (16.7)	50 (51) 31 (31.6) 7 (7.1)	69 (39.2) 65 (36.9) 39 (22.1)	0.004
Clinical Stage of urothelial BCa, *n* (%) Very high risk NMIBC MIBC	36 (14) 219 (86)	11 (12.2) 79 (87.8)	25 (15.2) 140 (84.8)	0.52
NAC for urothelial MIBC, *n* (%) Yes No	158 (72) 61 (28)	41 (52) 38 (48)	117 (83.5) 23 (16.4)	<0.001
Pathological T-stage, *n* (%) pT0 pTa–Tis–T1 pT2 pT3–T4	64 (23.3) 47 (17) 54 (19.7) 109 (40)	19 (19.3) 16 (16.3) 20 (20.4) 43 (43.8)	45 (25.5) 31 (17.6) 34 (19.3) 66 (37.5)	0.6
Pathological N-stage, *n* (%) pN0 pN1 pN2 pN3	210 (76.6) 22 (8) 37 (13.5) 5 (1.8)	74 (75.5) 9 (9) 12 (12.2) 3 (3)	136 (77.2) 13 (7.3) 25 (14.2) 2 (1)	0.6
Number of positive LNs, median (IQR)	3 (1–5)	3 (1–4.5)	2.5 (1–5)	0.9
Concomitant CIS, *n* (%)	74 (27)	28 (28.5)	46 (26.1)	0.4
Histology, *n* (%) Urothelial Non-urothelial	255 (93) 19 (7)	90 (92) 8 (8)	165 (93.7) 11 (6.3)	0.5

### LN yield and adequacy of PLND

The LN yield and adequacy of PLND are depicted in Table 2. The median LN yield was significantly higher in the patients who underwent R-PLND compared to those who underwent L-PLND. This difference was seen in both the S-PLND time period (median 16 LNs for R-PLND vs 11 for L-PLND, *P* < 0.001) and the current E-PLND period (median 19 LNs for R-PLND vs 14 for L-PLND, *P* < 0.001). Also, a significantly higher proportion of patients in the R-PLND group had an adequate PLND compared to the L-PLND group. A total of 64 (23.3%) patients had positive LNs on final histopathology, with 24 (24.4%) in the L-PLND group and 40 (22.7%) in the R-PLND group. The median number of positive LNs in the two groups was comparable (3 in the L-PLND group vs 2.5 in the R-PLND group, *P = *0.9).

**Table 2.** LN yield and adequacy of PLND in 274 patients who underwent either L- or R-PLND from January 2007 to July 2019

Variable	Overall	L-PLND	R-PLND	*P*
LN yield, median (IQR) Overall S-PLND (2007–2011) E-PLND (2012–2019)	16 (10–22) 12 (8–16) 18 (13–24)	11 (8–15) 11 (8–15) 14 (7–21)	19 (13–25) 16 (10–20) 19 (14–25)	<0.001 <0.001 0.009
Adequate PLND, *n* (%) S-PLND (2007–2011) E-PLND (2012–2019)	70 (68) 109 (63.7)	45 (61.6) 9 (36)	25 (83.3) 100 (69)	0.03 <0.001

### Predictors of an adequate PLND

We evaluated factors associated with an adequate PLND using univariable and multivariable logistic regression analyses (Table 3). The surgical approach to PLND (robot-assisted vs laparoscopy) was the only variable that was significantly associated with an adequate PLND on both univariable (odds ratio [OR] 1.860, 95% CI 1.114–3.105; *P* = 0.01) and multivariable (OR 2.109, 95% CI 1.222–3.641; *P* = 0.007) analyses. Other variables which included age, BMI, use of NAC and pathological T-stage were not found to be significant predictors of an adequate PLND on univariable analysis.

**Table 3.** Uni- and multivariable logistic regression analysis for prediction of an adequate PLND

Variable	Univariable analysis		Multivariable analysis	
	OR (95% CI)	*P*	OR (95% CI)	*P*
Age, years	0.970 (0.913–1.102)	0.08	0.962 (0.914–1.298)	0.1
BMI, kg/m^2^	0.992 (0.936–1.051)	0.8	0.984 (0.956–1.012)	0.9
NAC (yes vs no)	1.301 (0.783–2.161)	0.3	1.043 (0.605–1.797)	0.8
R- vs L-PLND	1.860 (1.114–3.105)	0.01	2.109 (1.222–3.641)	0.007
pT3–4 vs pT0–2	1.038 (0.582–1.851)	0.2	0.914 (0.536–1.506)	0.7

## Discussion

Pelvic LN metastasis is the single most important prognosticating factor following RC for bladder cancer and also guides further management decisions for use of adjuvant chemotherapy. Current cross-sectional imaging, although improving rapidly, has only a 48–87% sensitivity and similar specificity to detect pelvic LN metastasis and cannot detect micro-metastasis; while the role of fluorodeoxyglucose positron-emission tomography/CT is still unestablished [1]. PLND during RC is the most accurate method for LN staging. In an attempt to reduce perioperative morbidity, minimally invasive RC, either laparoscopic or robot-assisted, is being increasingly adopted world-wide [25]. Although there are multiple series comparing open RC vs either LRC or RRC with PLND, data with direct comparison between LRC and RRC are scarce. In fact, the only prospective evidence on this subject comes from the CORAL trial, which had only 20 patients in each arm [18]. Our present series provides one of the largest retrospective evidences comparing the outcomes of R- and L-PLND.

In our present series, patients in the R-PLND cohort had a higher ASA score and ACCI score. Although no definite explanation can be offered for this, it could probably be a result of a higher incidence of comorbid patients presenting to us in the latter part of the study period when the number of robot-assisted procedures exceeded the laparoscopic ones. Similarly, the use of NAC was higher in the R-PLND group, which can be explained by the fact that the NAC utilisation at our centre has grown substantially over time and so has the use of the robotic approach for RC with PLND. The ergonomic ease of performing a RRC was probably the main factor contributing to the shift from LRC to RRC.

While there has been much debate about the optimal extent of PLND, LN yield has been used as a surrogate marker of an adequate PLND. This parameter also allows comparison across various reports. The median number of LNs removed during a PLND ranges from 9–31 across various reports [6,10,12,16,21,26–29]. This difference may be attributed to the different PLND templates adopted in each study; with studies with a more extensive template reporting a higher LN yield. Also, the use of terminology regarding ‘standard’, ‘extended’ and ‘super-extended’ PLND is not consistent in literature. Our present results showed a median yield of 12 LNs using a standard template and 18 LNs with an extended template, comparable to previous reports

We found a significant difference between L- and R-PLND LN yields in our present series; in both the standard and extended templates. A recent meta-analysis by Feng et al. [30] evaluated four previous studies for LN yield and showed a significantly higher LN yield with RRC compared to LRC. The technical challenge associated with a LRC may hinder an optimal clearance of the pelvic nodal basins, thus leading to a lower LN yield. On the other hand, the dexterity offered by the robotic system allows easier access to the entire template of a S- and E-PLND. The large retrospective series included in the meta-analysis reported similar LN yields between L- and R-PLND (median 17 for L-PLND vs 18 for R-PLND) [19]. However, the PLND template used in the study was not defined. The CORAL trial was the only randomised prospective series comparing LRC and RRC and it too reported similar yields (mean 15.5 for L-PLND vs 16.3 for R-PLND) [18]. But, the sample size of 20 patients in each arm seems inadequate to draw robust conclusions.

There is no consensus about what constitutes an ‘adequate’ PLND to improve staging and oncological outcomes. Some authors have opined that completeness of resection within a selected template, rather than the total LN yield, is more important [31,32]. These studies analysed the LN yields in different institutes for the same templates. They did find discrepancies between the LN yields at different centres, but these were attributed to differences in the way the LNs were submitted for histopathology and also the pathological processing of the specimens. Although there can be no argument against the importance of a meticulous complete dissection in a selected template, the minimum number of LNs removed is probably the only parameter to objectively asses completeness of a PLND. Several authors have evaluated the oncological outcomes depending on the number of LNs removed and a general agreement suggests that at least 10 and 16 LNs should be removed in a S- and E-PLND, respectively [7,9,20,22]. We found that R-PLND resulted in a significantly higher probability of performing an adequate dissection than L-PLND, for both the S- and E-PLND templates. This result is likely a derivation of our previous result of a higher LN yield in the R-PLND group. We did not assess the impact of histopathological processing methods on the LN yield. But there is probably a need to standardise the method of submission of LN packets and their histopathological processing so that LN yields across various series can be compared without bias.

Besides the template of the PLND, several other factors have been implicated by some studies to impact the LN yield; similarly, other studies have denied their association. These factors include patient age, BMI, use of NAC and the pathological T-stage of the tumour. We evaluated these factors and also the variable of surgical approach to PLND (robot-assisted vs laparoscopic) in a multivariable logistic regression model to predict the outcome of an adequate PLND. We found that performing R-PLND instead of L-PLND was the only significant variable predicting an adequate PLND, both in the univariable and multivariable analysis. To the best of our knowledge, this represents the only evidence evaluating surgical approach to PLND as an independent predictor of an adequate PLND.

We do acknowledge certain limitations of our present study. Firstly, the study design was retrospective and thus subject to selection bias. However, the initial couple of years in the study period had exclusively L-PLND patients, while only R-PLND has been performed in the last 4 years. This would have reduced the selection bias to a certain extent. Secondly, we did not analyse the oncological benefit of achieving a higher LN yield or performing an adequate PLND. Despite these limitations, our present results provide the largest available evidence to support RRC with PLND over a LRC with PLND for achieving a higher LN yield and a greater probability of performing an adequate PLND. The ultimate aim is to stage patients more accurately after their surgery in order to be able to better stratify their prognosis and chose adjuvant treatments.

## Conclusion

R-PLND leads to a higher LN yield compared to L-PLND for both the S- and E-PLND templates. Also, with R-PLND there is a greater chance of removing the minimum defined number of LNs and thus achieving an adequate PLND. The surgical approach to PLND (robot-assisted vs laparoscopic) is an independent predictor of an adequate PLND. Thus, the robot-assisted approach would lead to more accurate staging following RC with PLND.
